# The effect of a mindfulness based-group psychotherapy on stress parameters in schizophrenia spectrum disorders

**DOI:** 10.1192/j.eurpsy.2025.720

**Published:** 2025-08-26

**Authors:** M. Zierhut, N. Bergmann, S. Koop, I. Hahne, J. Kraft, A. Braun, T. M. T. Ta, M. Bajbouj, N. Thomas, P. Chadwick, S. Ripke, E. Hahn, K. Böge

**Affiliations:** 1 Clinic for Psychiatry and Neurosciences, Charite - Universitätsmedizin Campus Benjamin Franklin; 2 Clinic for Psychiatry and Neurosciences, Charite - Universitätsmedizin Campus Mitte, Berlin, Germany; 3 Swinburne University of Technology, Melbourne, Australia; 4 University of Bath, Bath, United Kingdom

## Abstract

**Introduction:**

Recent studies have demonstrated the positive effects of mindfulness-based interventions on stress reduction in healthy individuals. Individuals with schizophrenia spectrum disorders (SSD) often experience elevated stress levels due to multiple factors. According to literature, plasma and saliva levels of oxytocin (OXT) and cortisol can serve as biological stress markers. However, the interaction between mindfulness, stress, and the oxytocinergic system in SSD remains unexplored.

**Objectives:**

This exploratory study investigates the impact of mindfulness-based group therapy (MBGT) on biological stress parameters, including OXT and cortisol levels in plasma and saliva, and changes in psychological stress parameters.

**Methods:**

A blinded, randomized, and controlled study was conducted. Participants were assigned to either MBGT with four weekly sessions in addition to treatment as usual (MBGT+TAU) or only treatment as usual (TAU). Venous blood and saliva samples were collected before and after the MBGT sessions to determine OXT and cortisol levels. Self-reported questionnaires measured stress via visual analogue scales before and after the MBGT sessions.

**Results:**

A total of 48 outpatients with SSD received either MBGT+TAU (n=25) or TAU (n=23). Analyses revealed a significant reduction in subjective stress levels during each MBGT session. After the MBGT sessions, a significant reduction in cortisol levels was observed, which correlated with the reduction in subjective stress experience. During the first session, oxytocin levels significantly increased in the saliva. However, in the last session, there was a significant decrease in oxytocin levels in both blood and saliva. Additionally, the MBGT+TAU group showed significantly lower OXT plasma levels at the end of the intervention compared to the TAU group.

**Image 1:**

**Figure:**
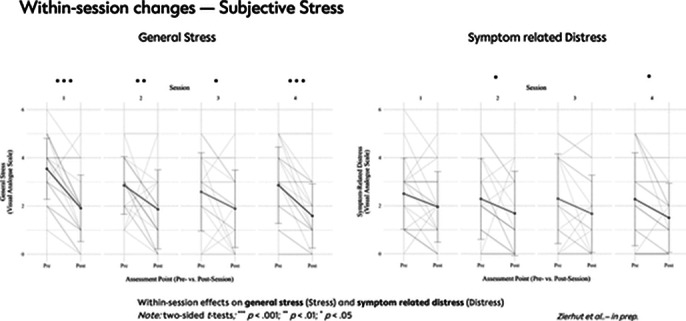

**Image 2:**

**Figure:**
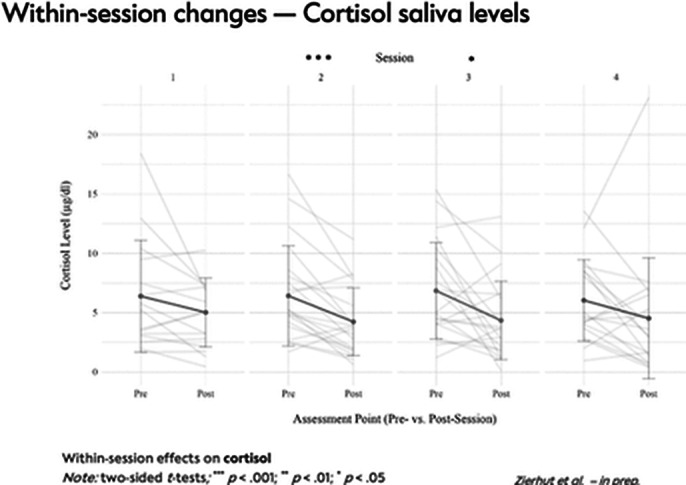

**Image 3:**

**Figure:**
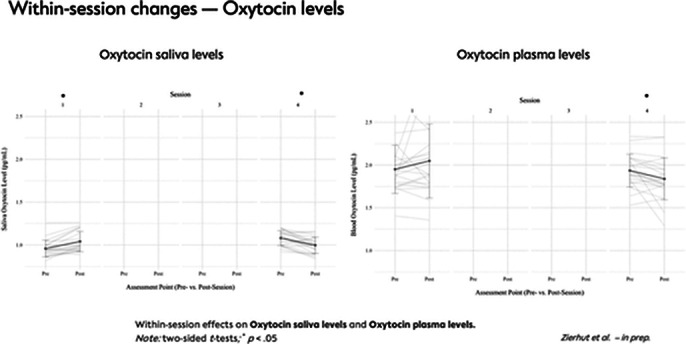

**Conclusions:**

The outcomes of this study provide insights into the potential effects of mindfulness on biological and psychological stress parameters in SSD. Consistent with recent research, we found significant effects on subjective stress and changes in oxytocin and cortisol levels throughout the MBGT intervention. A fully powered trial is needed to determine the efficacy of these findings.

**Disclosure of Interest:**

None Declared

